# Frequency dependence prediction and parameter identification of rubber bushing

**DOI:** 10.1038/s41598-022-04839-2

**Published:** 2022-01-17

**Authors:** Guang Li, Liguang Wu, Shuyu Zhang, Fang Liu

**Affiliations:** 1 CATARC Automotive Test Center (Tianjin) Co., Ltd., Tianjin, 300300 China; 2 China Automotive Technology and Research Center Co., Ltd., Tianjin, 300300 China; 3 School of Artificial Intelligence and Automation, HUST, Wuhan, 430074 China; 4 School of Mechanical Engineering, Hebei University of Technology, Tianjin, 300300 China

**Keywords:** Engineering, Mechanical engineering

## Abstract

Affected by frequency, amplitude and some other factors, the dynamic mechanical properties of rubber bushing are nonlinear. In order to study the frequency dependence of the rubber bushing, a BP neural network optimized by genetic algorithm (GA-BP neural network) is applied to predict the dynamic stiffness and loss factor under frequency of 61–100 Hz. The training data refers to the test data under frequency of 1–60 Hz. And the algorithm is demonstrated by the elastomer test of rubber bushing under amplitudes 0.2 mm, 0.4 mm and 0.6 mm. The results show that the prediction error of dynamic stiffness is less than 1%, and the prediction error of loss factor is less than 3%. In order to apply the predicted results to the software for simulation, a five-parameter mathematical model (FPM) consisting of three elastic elements and two damping elements is developed, and the model parameters are identified by least squares method. According to the fitting results and test data, the fitting error of dynamic stiffness is less than 2%, and the fitting error of loss factor is less than 3%. The GA-BP neural network and FPM model predict the dynamic mechanical behaviour of rubber bushing without the performance of iterative experiments and the incurrence of a high computational cost, making it applicable to analyze full-size vehicles with numerous rubber bushings under various vibration load conditions.

## Introduction

As connections among components, rubber bushings, which can reduce NVH (noise, vibration, harshness) performance and make up for manufacturing tolerances, play an important role in automotive, aviation and other fields^1^. The complex mechanical behaviour of rubber bushing is expressed as elasticity and damping^2^, which can be described by stiffness and loss factors respectively^3^. Due to the influences of factors such as frequency, amplitude and temperature, variations of stiffness and loss factor are nonlinear. Therefore, research on the dynamic mechanical behaviour of rubber bushing which is significant to the ride comfort, handling stability, and NVH of the vehicle becomes an industry problem.

The rubber bushing is composed of rubber vulcanized to metal material^4^. Due to the nonlinear mechanical character of rubber material, it becomes a topical issue in the industry to establish predictive models and mathematical models that accurately describe the mechanical property^5^.

In order to research the frequency dependence and amplitude dependence of rubber bushing, physical models including elastic elements and damping elements are usually applied. The relationship between force and displacement or stress and strain in the models is derived by mathematical formulas. A model consisting of three elements: a parallel spring element, an amplitude-dependent element and a frequency-dependent element is developed to study amplitude and frequency dependence by Jun^6^. The model can be accurately and efficiently used for investigating the vehicle dynamics performances in the early phases of vehicle development. Based on measurement observations, a model depicting the axial and radial stiffness is established by García Tárrago^7^ to study the frequency and amplitude dependence of the rubber bushing. It’s simulation results of loss factor are slightly different from the test results. Subsequently, a model describing the torsion direction of the rubber bushing is utilized in the same way^8^. Eren^9^ develops a simplified frequency-dependent transfer function model which is demonstrated on a heavy commercial truck. The results show the improvement of the accuracy of the methodology compared to the Voigt model. Olsson^10,11^ describes frequency and amplitude dependence by adding integer derivatives to the stick–slip friction component and it is similar to Gil-Negrete^12^. Through Feng’s^13^ and Yu’s^14^ researches, the least square method is applied to identify the parameters of the Kelvin-Voigt model, Berg model and ASCL model, then the fitting exactitude of the three models to the mechanical behaviour of rubber bushing is compared. These scientific achievements provide theoretical support for the later study on the rubber bushing. For example, a semi-empirical parameterized rubber bushing model incorporating Dzierzek-based nonlinear elastic element model, fractional derivative viscoelastic model and Berg smooth friction model is established by Yu^15^, however, in the frequency range of 1–100 Hz, the maximum fitting error of the model's lag angle is 10.43%. A hybrid approach based on finite element analysis and empirical modeling is proposed by Lee^16^. In the finite element analysis, the hysteresis of the rubber bushing is obtained by the super elastic-elastoplastic model and the superposition method; in the empirical model, the spring, fractional derivative and friction component are employed to obtain the dynamic stiffness of a wide range of excitation frequency and amplitude. Guillaume^17^ establishes a model to describe the time-domain nonlinearity of rubber bushing, and adjusts the coefficients of the model through multiple test data. A dynamic model technique is proposed by Lee^18^. The performance of the developed technique in dynamic analysis is illustrated with two four-degree-of-freedom systems by comparing the results with those of the conventional Voigt model.

The temperature and preload dependence of rubber bushing are researched by transforming the amplitude and frequency models. According to Peter^19^, a model representing the temperature dependence of the bushing is established. Concerning the temperature dependency, it can be seen that the rise of temperature reduces the influence of frequency changes and amplitude changes. With rising temperature, the polymer chain movement is eased and a more frequent displacement can be accommodated with less resistance. He^20^ applies the optimized KVBC (Kelvin-Voigt and Bouc-wen) theoretical model to the calculation of multi-body dynamics through the secondary development of Adams, and extends the preload and temperature to the model^21^. As a result, when the preload rises, the stiffness increases, while the damping angle decreases.

Currently, based on the test data of low and medium frequency, most of the models are proposed to describe the frequency dependence of rubber bushing. While few studies on the prediction of the mechanical behaviour of rubber bushing at high frequency are conducted. Predicting the mechanical performance of rubber bushing reduces the test cycle and cost. It improves the precision of multi-body dynamics models, and contributes to predicting handling stability and ride comfort of the vehicle.

Genetic algorithm, developed by John Holland in 1975, is a metaheuristic inspired by the mechanisms of evolution. And it has been applied to many fields in search and optimization that belongs to the large class of evolutionary algorithms. The principle of the genetic algorithm is "natural selection, survival of the fittest" in the process of natural evolution, which generates next generation solutions through operations such as duplication, crossover, and mutation. The nonlinear problems are difficult for traditional method, but genetic algorithm shows advantages when solving these questions.

In this paper, a back propagation neural network optimized by genetic algorithm which is defined as GA-BP is applied to predict the frequency dependence of the rubber bushing. The prediction algorithm is demonstrated by experimental data of rubber bushing under different amplitudes. Compared with the mathematical model, the application of GA-BP neural network can more accurately predict the high-frequency fluctuation of the rubber bushing and reduce the complicated parameter adjustment process. In addition, a five-parameter mathematical model (FPM) is developed to fit the forecast data, and the parameters of the FPM model are identified.

## Prediction of frequency independence of rubber bushing

**Figure 1:**
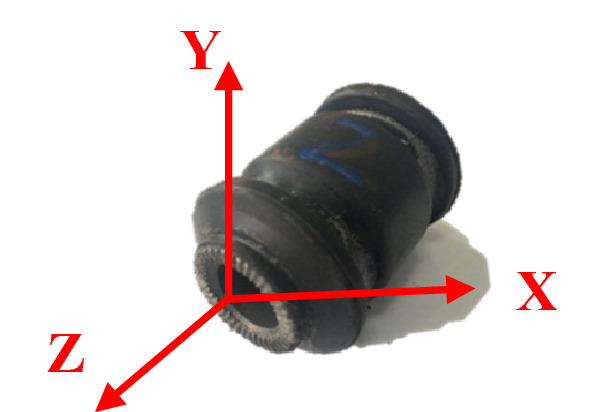
Rubber bushing.

**Figure 2:**
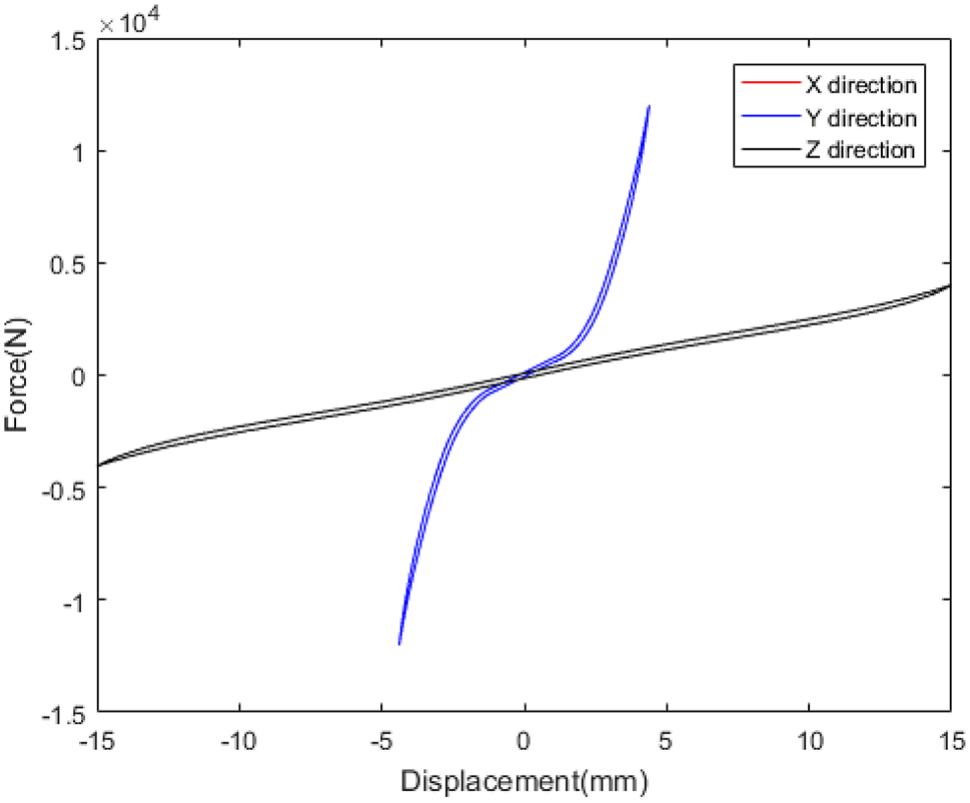
Translational stiffness in x/y/z direction.

**Figure 3:**
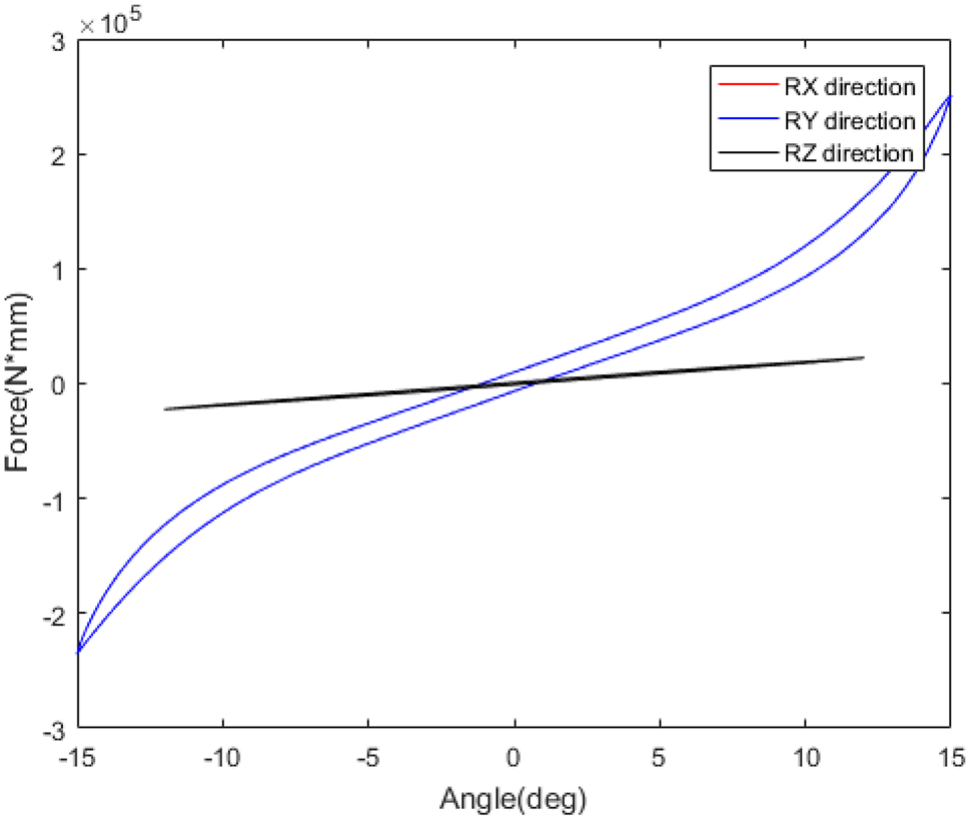
Torsional stiffness in x/y/z direction.

**Figure 4:**
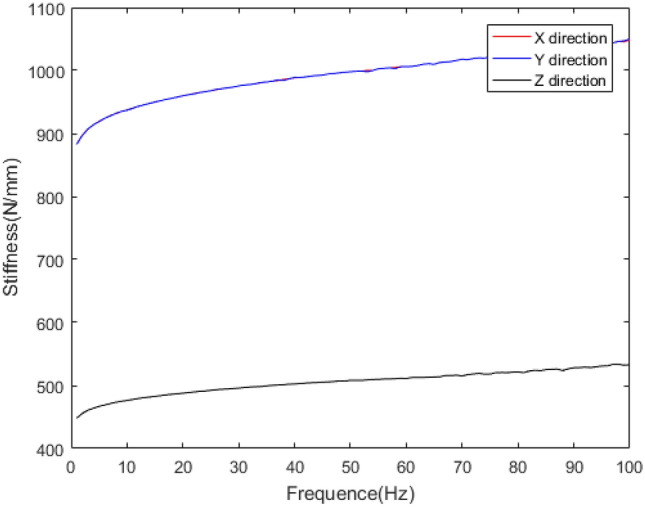
Relationship between translational stiffness in x/y/z direction and frequency.

**Figure 5:**
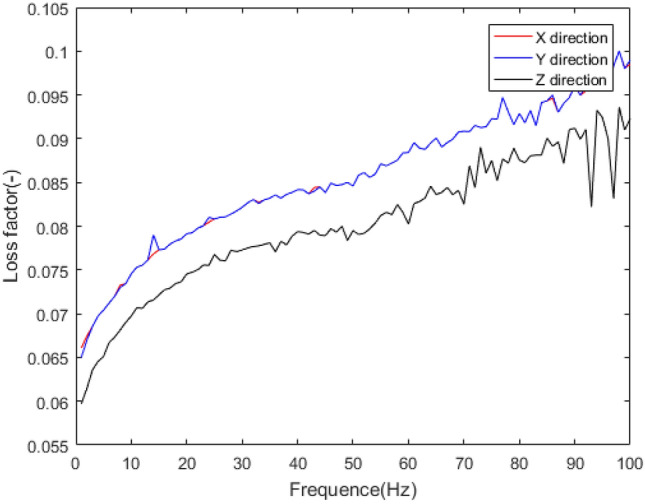
Relationship between translational loss factor in x/y/z direction and frequency.

**Figure 6:**
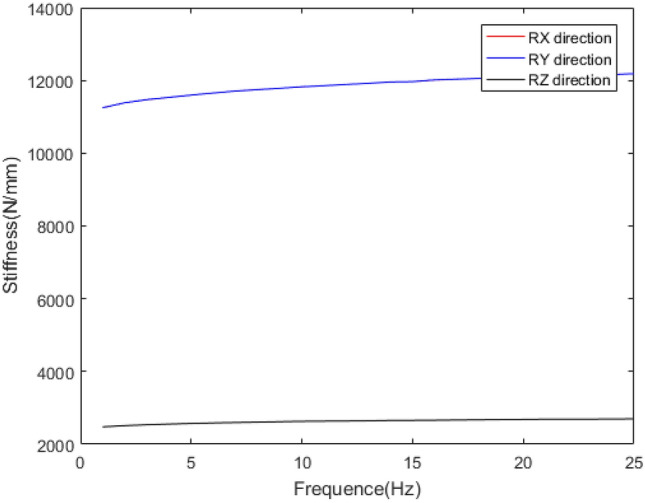
Relationship between torsional stiffness in x/y/z direction and frequency.

**Figure 7:**
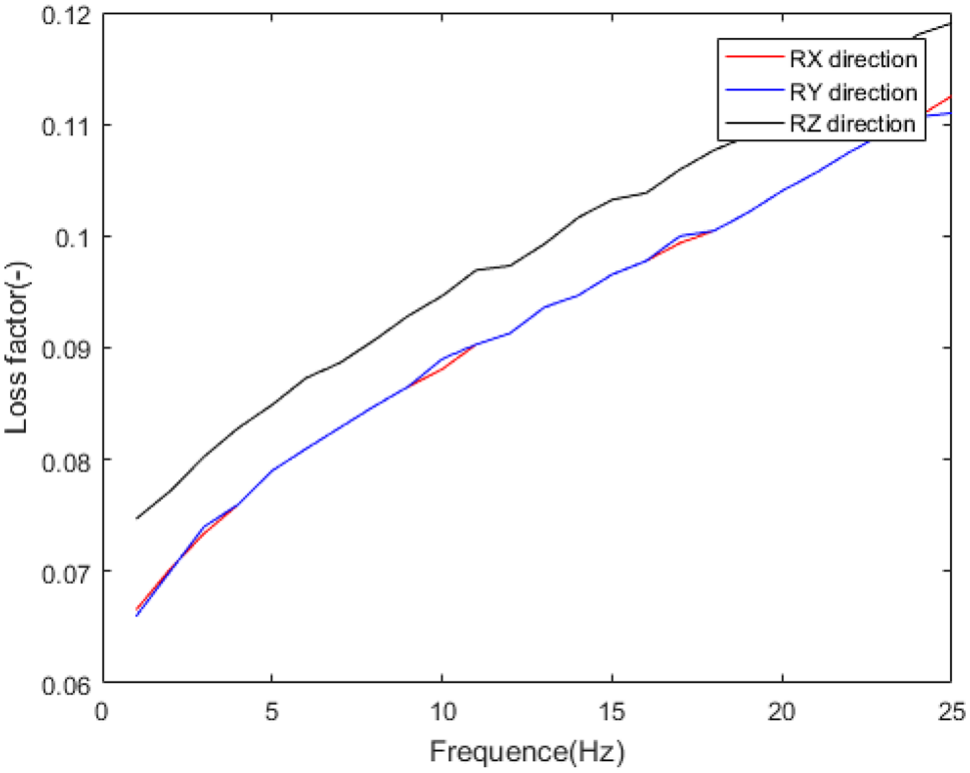
Relationship between torsional loss factor in x/y/z direction and frequency.

The mechanical behaviour of rubber bushing is mainly manifested as force VS displacement (stiffness) and force VS velocity (damping), and the damping can be represented by loss factor. Referring to the literature^22^, a rubber bushing displayed in Fig. 1 is tested to analyze the mechanical properties in the six directions. And the test results are shown in Figs. 2, 3, 4, 5, 6, 7. The rubber bushing is a solid bushing, that is, the mechanical properties of radial X and Y directions are the same.

Rx/Ry/Rz represent the torsion direction of X/Y/Z respectively. Figures 2 and 3 illustrate the correlation between displacement and force in the six directions. The stiffness and loss factor in the six directions are shown in Figs. 4, 5, 6, 7. Due to the insufficient installation stiffness of the test bench, when the frequency exceeds 25 Hz, the stiffness in the torsional direction will produce obvious errors. The number of 25 samples is not enough to ensure the accuracy of data prediction. In addition, the mechanical behavior of rubber bushing has the similar changing trend in the six directions. Therefore the radial mechanical property of the translation direction is taken as an example to carry out the test and predictive analysis.

### Test of rubber bushing

**Figure 8:**
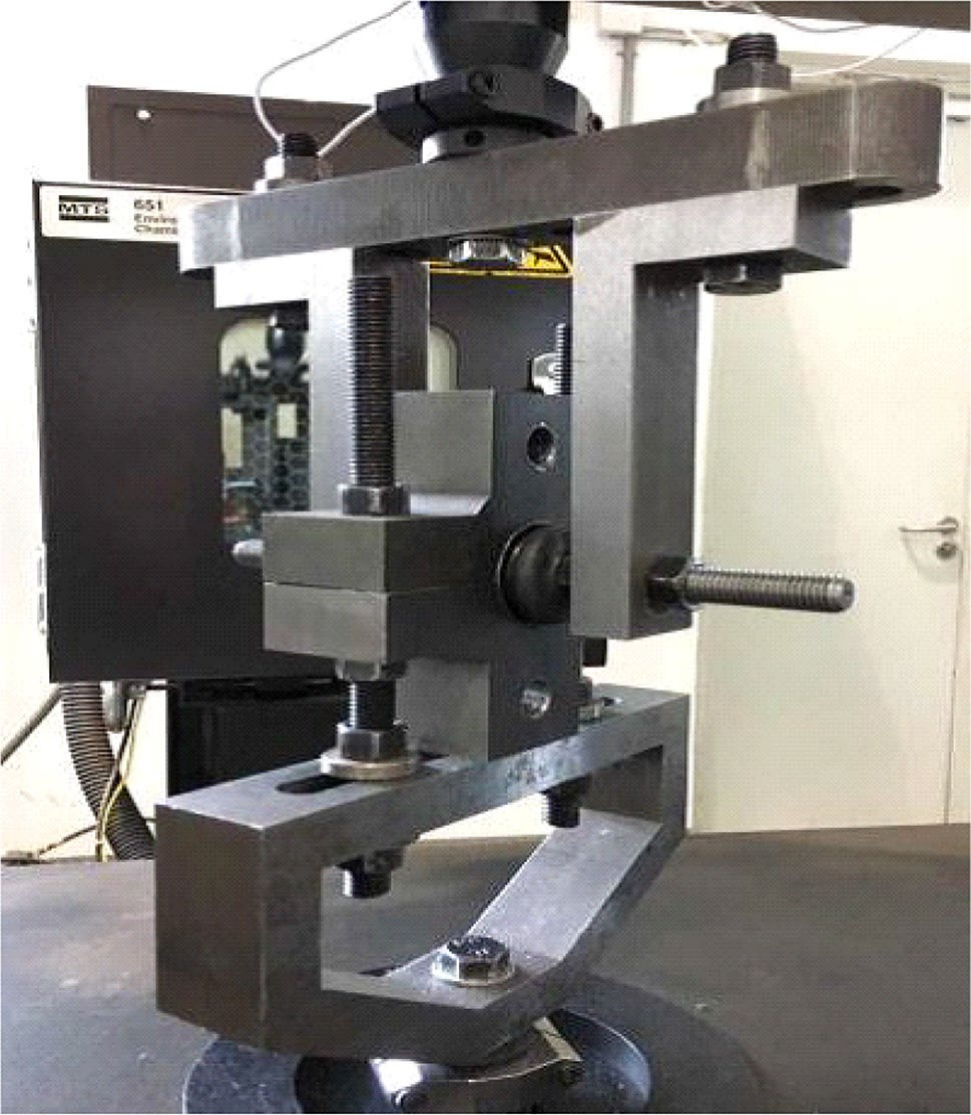
Elastomer test.

**Figure 9:**
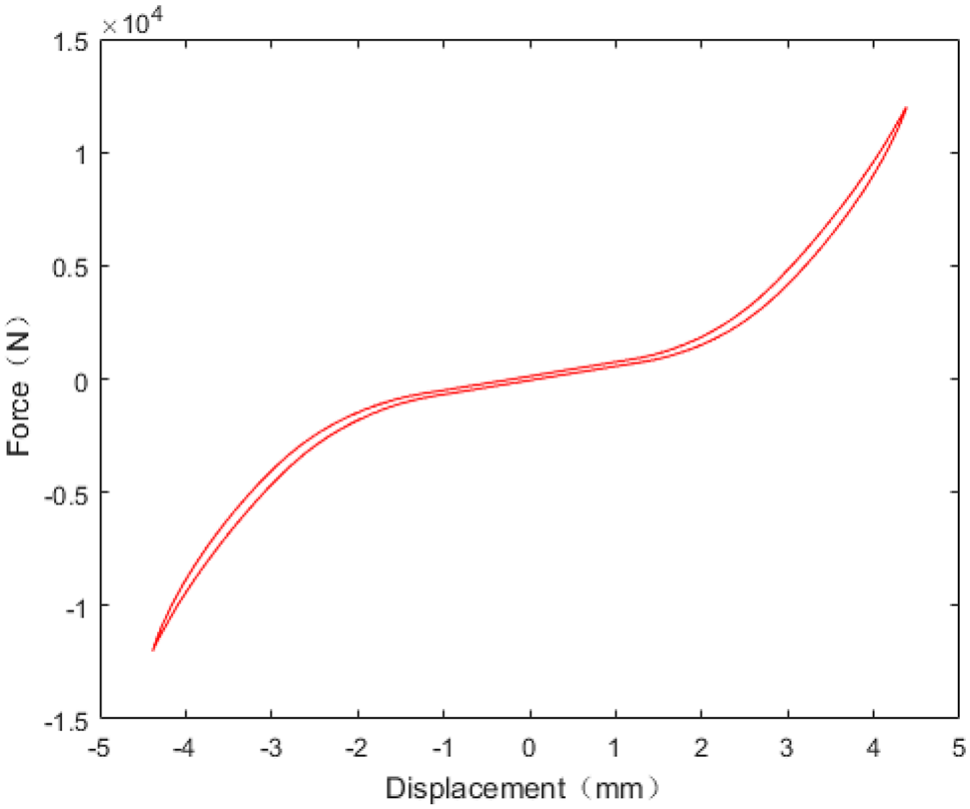
Radial stiffness of rubber bushing in static test.

**Figure 10:**
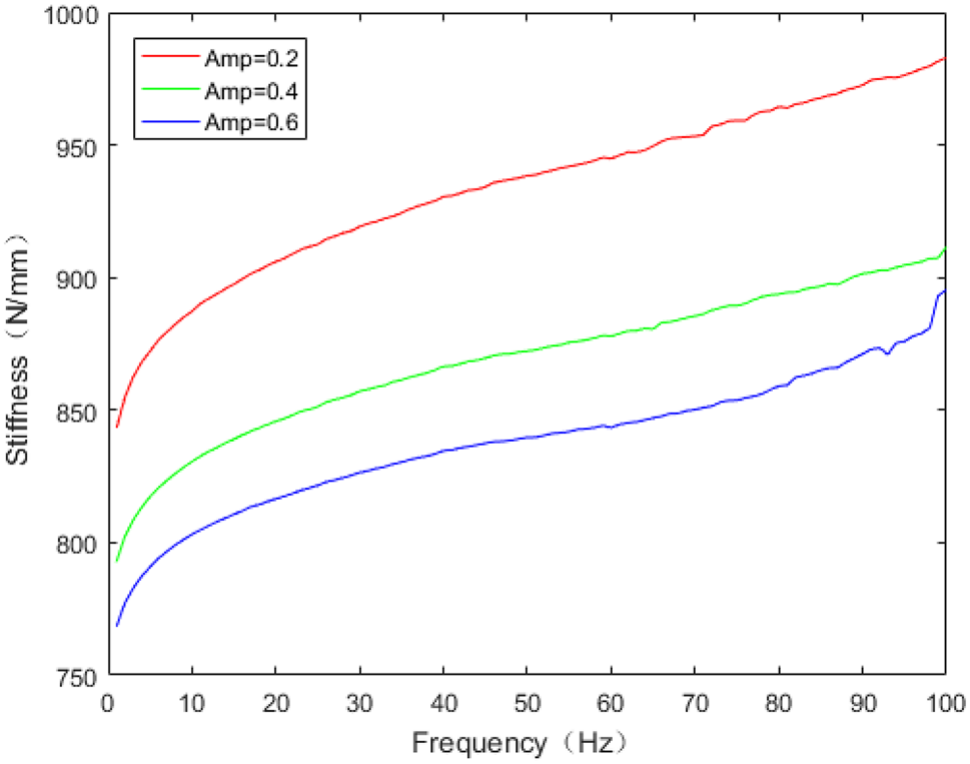
Relationship between radial stiffness and frequency in test.

**Figure 11:**
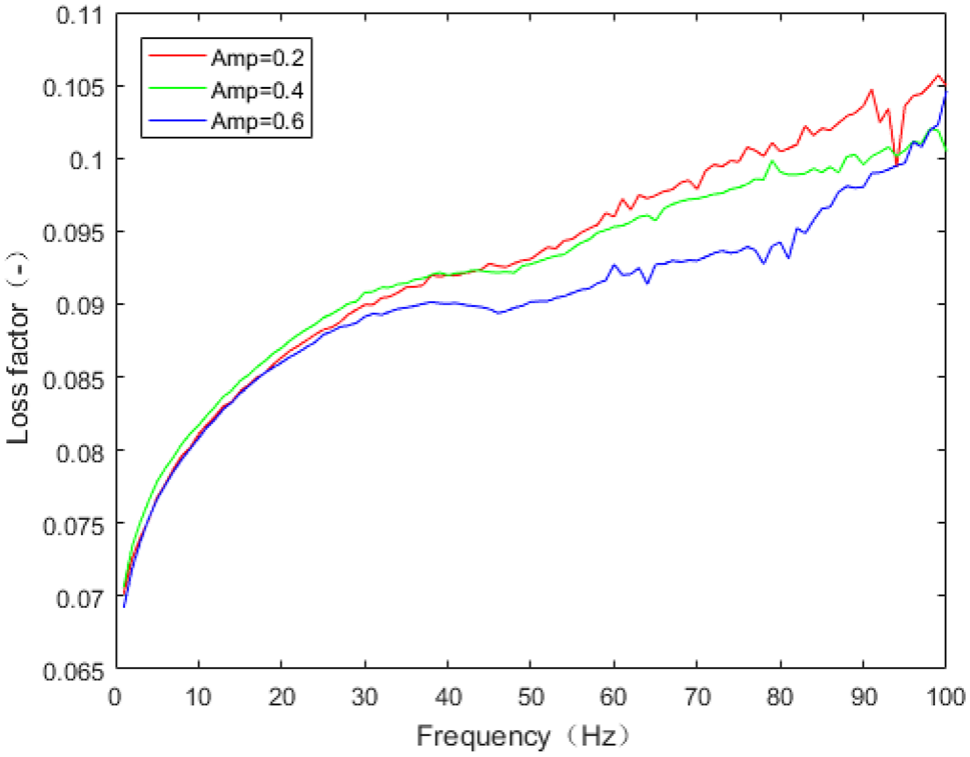
Relationship between radial loss factor and frequency in test.

The mechanical behaviour test of rubber bushing is divided into static performance test and dynamic performance test. The mechanical behaviors are described in Figs. 9, 10, 11. The test-bed is displayed in Fig. 8, and the parameters and settings of test are listed in Table 1.

**Table 1:** Test parameters and settings.

Parameters	Settings
Method	Uniaxial stretching
Equipment	MTS831.50
Temperature	23 ± 2 °C
Preload	2000 N
Tensile force	± 8000 N
Frequency	1:1:100 Hz
Amplitude	0.2, 0.4, 0.6 mm

The rubber is composed of chain molecules which have irregular shape and high entropy. When the rubber is stimulated, the chain structure changes constantly, resulting in obvious strain deformation and large entropy. The correlation between displacement and force under static test is illustrated in Fig. 9. While the displacement is less than 2 mm, the static stiffness is 855.1 N/mm and close to linear. While the displacement is greater than 2 mm, the static stiffness is 6253 N/mm and tends to be nonlinear. Obviously, the nonlinear stiffness is significantly greater than the linear stiffness.

The dynamic stiffness and loss factor are indicated to the dynamic behaviour of rubber bushing, which are shown in Figs. 10 and 11. Dynamic stiffness depends on the variation of stiffness with frequency and amplitude (represented by amp in the figures, unit: mm). Loss factor refers to the tangent of the lag angle between response force and excitation displacement. Due to the existence of intermolecular force, when the rubber is excited by external force, it shows obvious stress relaxation and creep under the influence of frequency and amplitude. As a result, when the amplitude remain constant, with the rising frequency, the dynamic stiffness and loss factor of the rubber bushing trend to increase. When the frequency is invariant, the greater the amplitude, the less the loss factor and the dynamic stiffness.

### Predicting the frequency dependence of rubber bushing with GA-BP neural network

Numerous sampling frequencies are used for testing to study the mechanical behaviour of rubber bushing, especially studying the NVH characteristic, which greatly increases the cost and cycle of experiment. In order to reduce cost and cycle, BP neural network is applied to predict frequency dependence of the rubber bushing. With the rising frequency, the prediction error become large. Therefore, the GA-BP neural network is utilized to predict the mechanical performance of rubber bushing with frequency in this paper.

**Figure 12:**
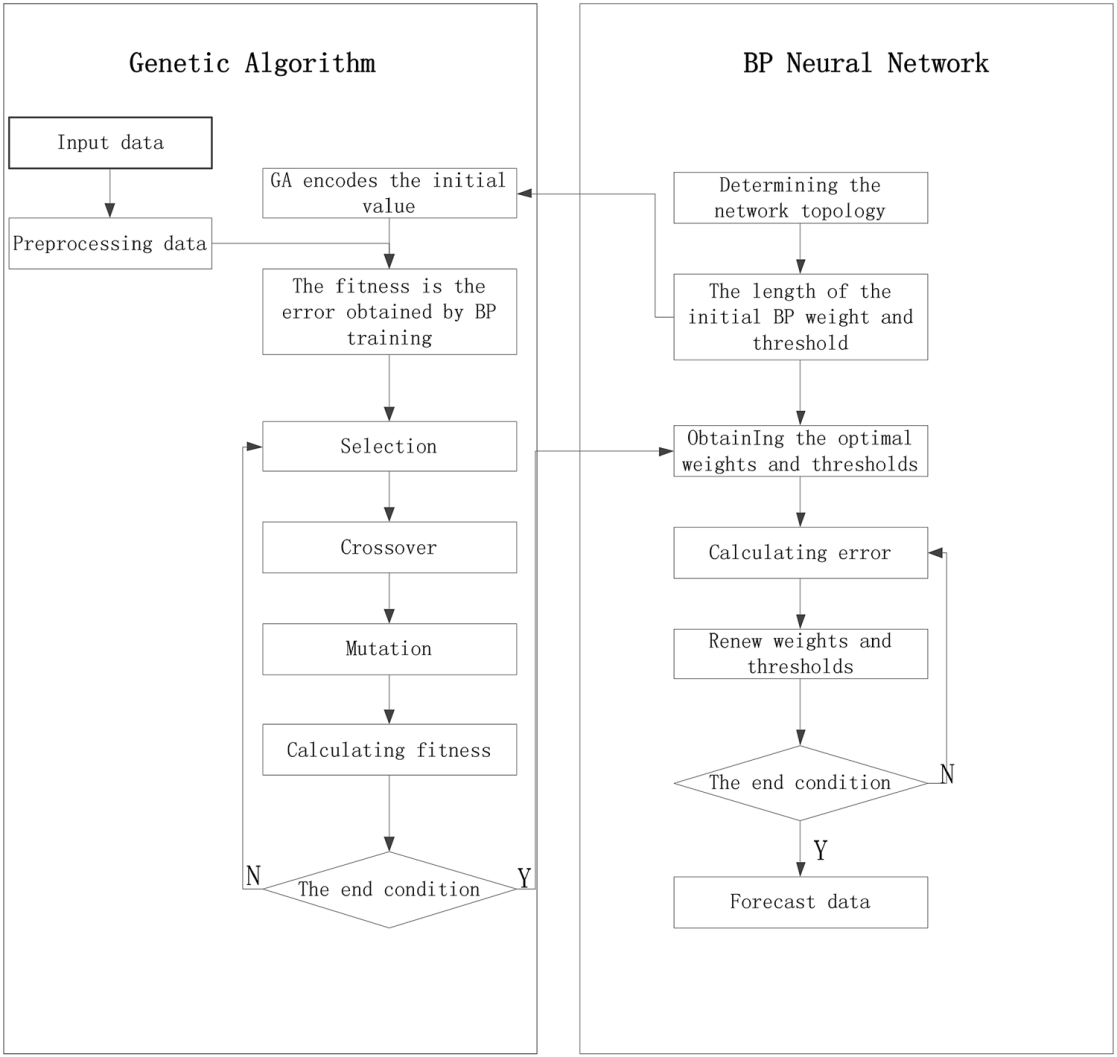
Topological structure of GA-BP neural network.

BP neural network is a multi-layer feedforward neural network, whose main feature expresses that the signal is forwarded and the error is back-propagated^23,24^. The steps to building the GA-BP neural network are as follows: firstly, according to the errors between predictive stiffness, loss factor and test results, the network weights and thresholds are adjusted to make the predicted results approach training data. Then, the optimal weights and thresholds corresponding to the selected optimal fitness individuals are assigned to the BP neural network so as to reduce the errors of predicted stiffness, loss factor. The topological structure of GA-BP neural network is sketched in Fig. 12. The settings of genetic algorithm and BP neural network are set out in Table 2.

**Table 2:** Settings of genetic algorithm and BP neural network.

Algorithm	Parameter	Value
BP neural network	Input neuron	2
	Hidden layer	5
	Output neuron	1
	Learning frequency with tutor	1–60
	Learning frequency without tutor	61–100
	Learning rate	0.1
Genetic algorithm	Number of iterations	150
	Population size	200
	Crossover	0.7
	Mutation	0.1

In genetic algorithm:

The fitness is calculated as follows:

1 $$ {\text{F = k}}\left( {\sum\limits_{i = 1}^{n} {abs(y_{i} - 0_{i} )} } \right) $$

where: *k* is the coefficient. n is the number of output nodes. *i* is the i-th node, y is the expected output, and o is the predicted output.

The selection method is computed as follows:

2 $$ {\text{p}}_{{\text{i}}} = k \div F_{i} * \sum \limits_{j = 1}^{N} {f_{i} } $$

where: *N* is the number of individuals in the population.

The selected crossover method:

3 $$ \left\{ \begin{gathered} {\text{a}}_{kj} = {\text{a}}_{{{\text{kj}}}} (1 - b) + a_{lj} b \hfill \\ a_{lj} = a_{lj} (1 - b) + a_{kj} b \hfill \\ \end{gathered} \right. $$

where: *a* is a chromosome. *b* is a random number between [0,1].

The selected mutation method:

4 $$ {\text{a}}_{{{\text{ij}}}} = \left\{ \begin{gathered} a_{ij} + (a_{ij} - a_{\max } )*f(g) \quad  r > 0.5 \hfill \\ a_{ij} + (a_{\min } - a_{ij} )*f(g) \quad  r \le 0.5 \hfill \\ \end{gathered} \right. $$

where: *f(g)* is defined as follows:

5 $$ {\text{f}}(g) = r(1 - g \div G_{\max } )^{2} $$

where: *g* is the current iteration number. *G*_*max*_ is the maximum evolution number. *r* is a random value between [0,1].

**Figure 13:**
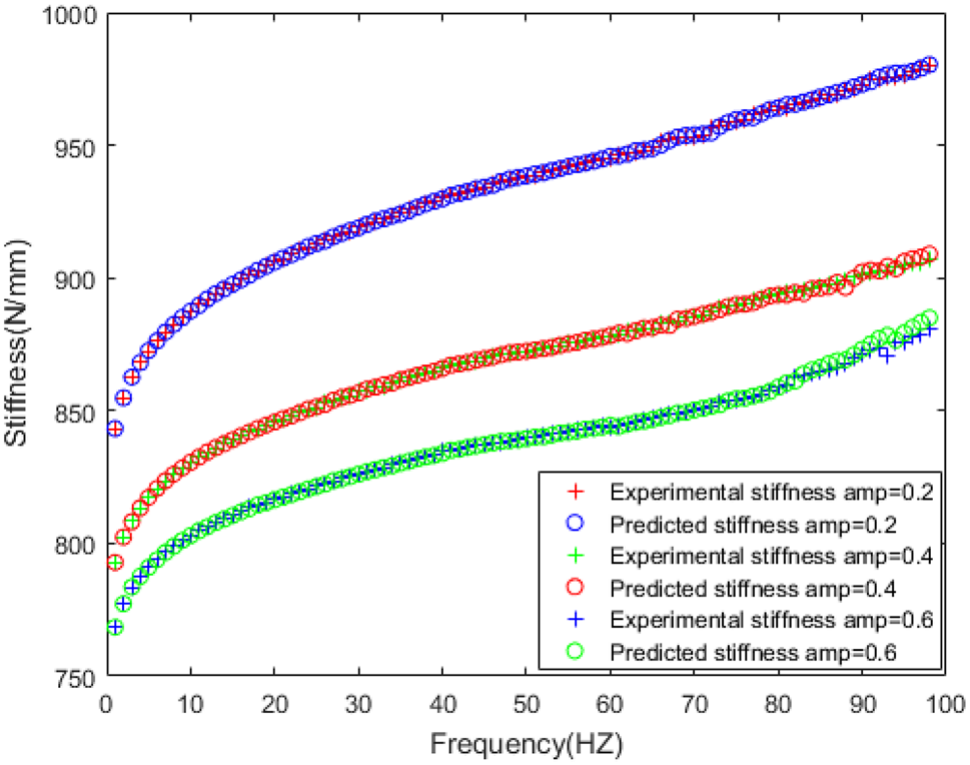
Comparison of prediction results and experimental results of dynamic stiffness under different amplitudes.

**Figure 14:**
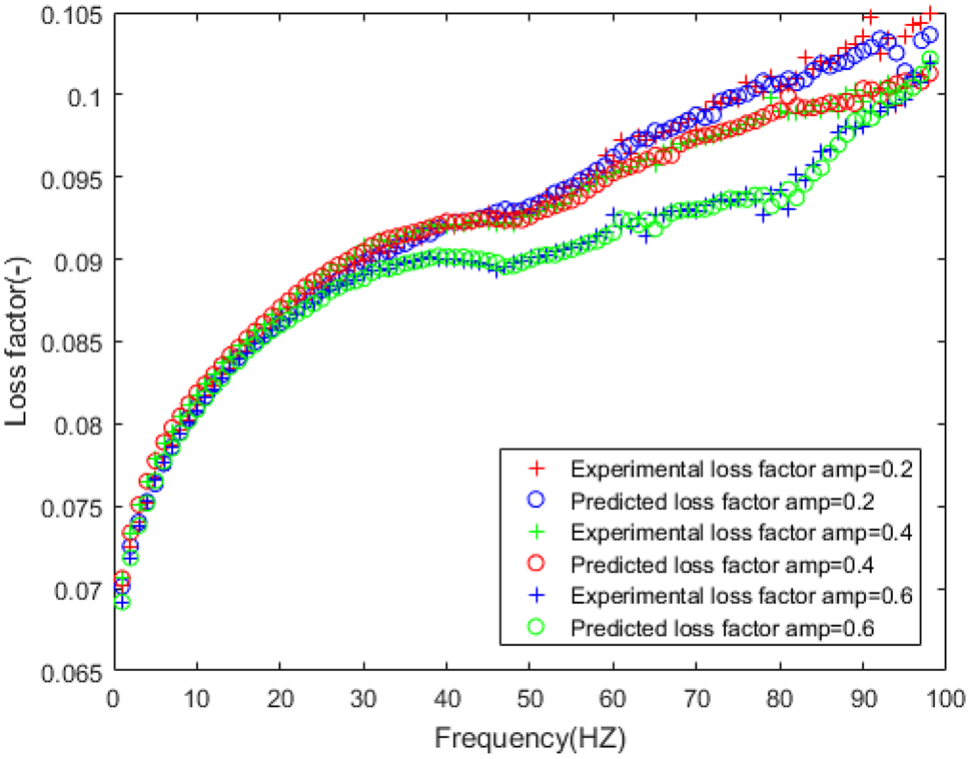
Comparison of prediction results and experimental results of loss factor under different amplitudes.

**Figure 15:**
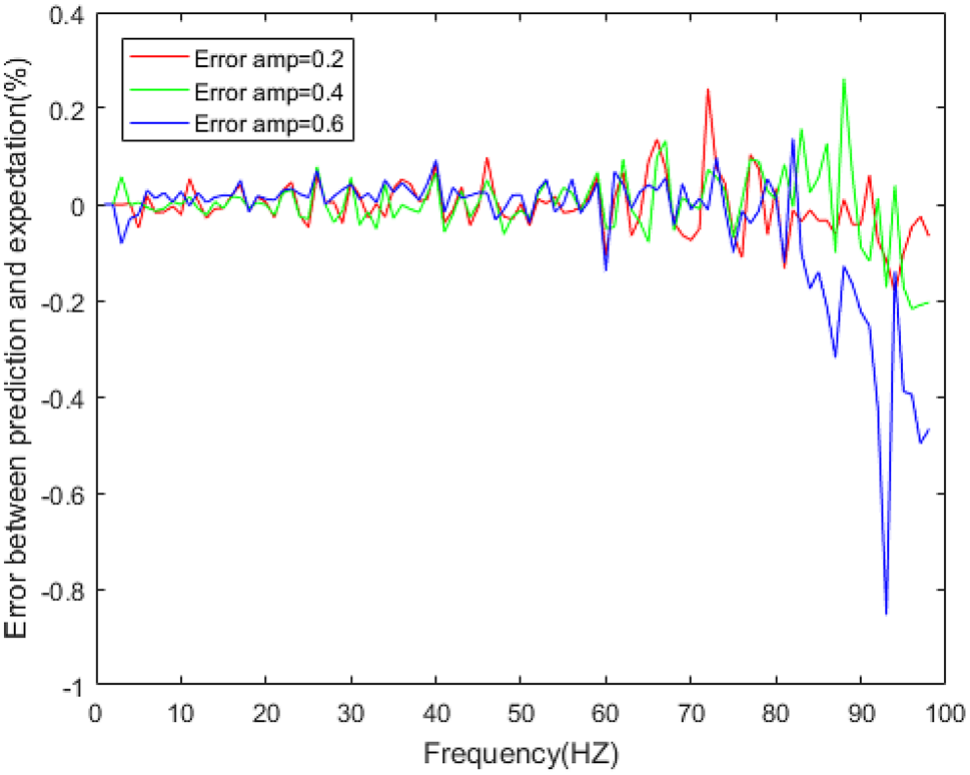
Errors between the prediction results and experimental results of dynamic stiffness under different amplitudes.

**Figure 16:**
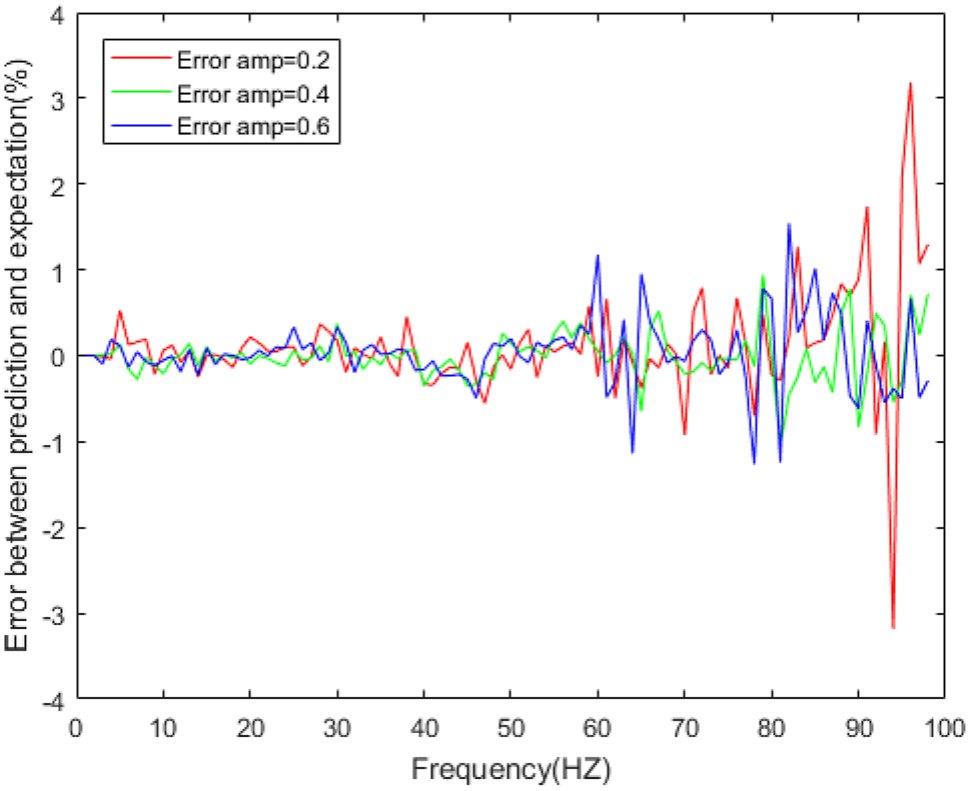
Errors between the prediction results and experimental results of the loss factor under different amplitudes.

According to the above fitness, selection, crossover, and mutation methods, the BP neural network is optimized to predict the frequency dependence of the rubber bushing. The prediction results of dynamic mechanical properties are sketched in Figs. 13, 14, 15, 16.

The dynamic stiffness prediction values and errors of the rubber bushing under amplitude 0.2 mm, 0.4 mm, and 0.6 mm are displayed in Figs. 13 and 15. The GA-BP neural network is applied to predict the dynamic stiffness under amplitude 0.2 mm, and the algorithm’s precision is verified by predicting the dynamic stiffness of rubber bushing under amplitude 0.4 mm, 0.6 mm. In the frequency range of 1–60 Hz, the forecast data is trained with the instructor, and the prediction error of dynamic stiffness is less than 0.2% under different amplitudes. When the frequency range is between 61 and 100 Hz, the forecast data is predicted without the instructor. In the high frequency range, especially above 90 Hz, during the rubber bushing experiment, the test bench inevitably produces small amplitude vibrations, which causes fluctuations in stiffness and loss angle changes. Generally speaking, the prediction error of dynamic stiffness is less than 0.8% under different amplitudes.

In Figs. 14 and 16, the loss factor prediction results and errors of the rubber bushing under amplitude 0.2 mm, 0.4 mm, and 0.6 mm are represented. In the frequency range of 1–60 Hz, the forecast data is trained with the instructor, and the prediction error of loss factor is less than 0.4% under different amplitudes. When the frequency range is between 61 and 100 Hz, the forecast data is predicted without the instructor, and the prediction error is less than 3% under different amplitudes.

The error analysis indicates that the GA-BP neural network accurately predicts the frequency independence of rubber bushing. In actual engineering, predicting the frequency independence of rubber bushing can not only reduce test cost and cycle, but also forecast the impact of rubber bushing mechanical behaviour on vehicle comfort and NVH performance.

The frequency independence of rubber bushing is predicted mainly by forecasting the value of the next frequency through the previous two values. While the frequency interval is small, there will be exponentially increased data and forecast time. In addition, in practical applications, the mathematical formulas are generally used to describe the mechanical characteristic of rubber bushing in multi-body dynamics software. Therefore, it is necessary to establish a mathematical model describing the mechanical behaviour based on the forecast data.

## Mechanical behaviour model of rubber bushing and parameter identification

The mechanical behaviour of rubber bushing includes static mechanical characteristic and dynamic mechanical characteristic. The constitutive models depicting static mechanical behaviour include Mooney-Rivlin model^25^, Neo-Hookean model^26^, Yeoh model^27^, Ogden model^28^ and some other models. The parameters which represent the material behaviour of the rubber bushing in these models have actual physical meanings, and the relationship between stress and strain is described by the models. Since the relationship between force and displacement of the rubber bushing is considered in the paper, a polynomial spring model can be employed to describe the static mechanical performance of the rubber bushing^29^. The model expresses the nonlinear relationship between stiffness and load displacement, but also displays the stiffness’s difference during forward and reverse loading. The polynomial spring model is calculated as

6 $$ {{F = a}}_{0} + a_{1} x + a_{2} x^{2} + \cdots + a_{n} x^{n} $$

where: *F* is the loading force of the rubber bushing, *x* is the radial deformation displacement of the rubber bushing, *a*_*1*_*, a*_*2*_*….a*_*n*_ are coefficients of the polynomial spring model.

The research on the dynamic behaviour of rubber bushing is usually divided into two methods: ellipse method and transfer function method. Due to its simplicity and intuition, transfer function method is extensively applied to describe the variation of the mechanical behaviour of rubber bushing with various impact factors. The transfer function method refers to fitting the experimental data of dynamic stiffness and loss factor by developing a physical model, for example, three-parameter Maxwell model^30^, K-V model^31^, Berg Model^32^, etc.

### Five-parameter mathematical model

**Figure 17:**
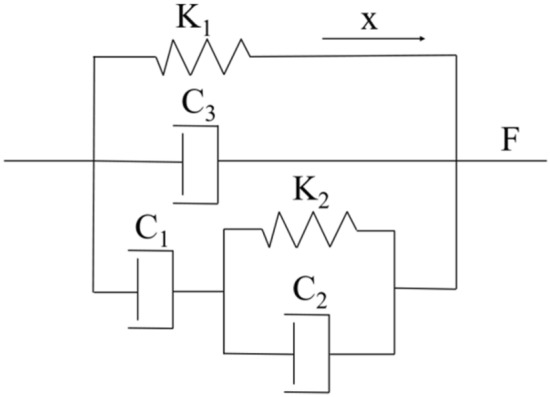
Five-parameter mathematical model.

When the influencing factors of rubber bushing are analyzed, the influencing factors are regarded as irrelevant. Based on the forecasted dynamic stiffness and loss factor data of rubber bushing, the FPM model is established to describe the dynamic behaviour of rubber bushing. Because amplitude dependence of rubber bushing is precisely fitted by the Bouc-wen model, the study about amplitude dependence is not conducted in this paper. In the FPM model illustrated in Fig. 17, the stiffness performance is expressed by spring elements, and the damping characteristic is expressed by damping elements.

The spring elements describing the stiffness performance of the rubber bushing are composed of *K*_*1*_, *K*_*2*_. The damping elements expressing the damping characteristic of the rubber bushing are composed of *C*_*1*_, *C*_*2*_, and *C*_*3*_. In detail, the spring element *K*_*2*_ and damping element *C*_*2*_ are connected in parallel, then connected in series with the damping element *C*_*1*_*.* The component model is united in parallel with the damping element *C*_*3*_ and the spring element *K*_*1*_ to form the five-parameter mathematical model.

The relationship between force and loading displacement in the model is following:

7 $$ \left\{ \begin{aligned} & {\text{F}}_{{1}} = K_{1} x \hfill \\ & F_{2} = K_{2} z + C_{2} \dot{z} = C_{1} (\dot{x} - \dot{z}) \hfill \\ & F_{3} = C_{3} \dot{x} \hfill \\ & F = F_{1} + F_{2} + F_{3} \hfill \\ \end{aligned} \right. $$

where: *x* is the loading displacement of the rubber bushing, *z* is the displacement of the spring element *K*_*2*_ and the damping element *C*_*2*_, *F* is the force on the rubber bushing, *K*_*1*_ is the static stiffness of the spring.

Equation (7) is derived:

8 $$ {\dot{z} } =\frac{1}{{1{ + }\beta }}\left( {\dot{x} - \frac{\alpha }{\gamma }z} \right) $$

where:$$\begin{array}{*{20}c} {\alpha  = \frac{{k_{1} }}{{K_{2} }}} & {\beta = \frac{{C_{1} }}{{C_{2} }}} & {\gamma = \frac{{C_{1} }}{{K_{1} }}} \\ \end{array}$$.

Incorporating Eq. (8) into Eq. (7) for Laplace transform:

9 $$ {\text{G}}(S) = \frac{X(S)}{{F(S)}} = \left[ {\frac{(1 + \beta )\gamma }{{\alpha K_{1} }}S + \frac{1}{{K_{1} }}} \right]*\frac{1}{{\frac{{(C_{1} + C_{3} )(1 + \beta ) - C_{1} \gamma }}{{\alpha K_{1} }}S^{2} + \frac{{(C_{1} + C_{3} )\alpha + (1 + \beta )\alpha K_{1} }}{{\alpha K_{1} }}S + 1}} $$

Transforming the frequency characteristics of Eq. (9):

10 $$ \left\{ \begin{aligned} & {\text{A}}(\omega ) = \left| {G(j\omega )} \right| = \sqrt {\left[ {U(\omega )} \right] + \left[ {V(\omega )} \right]} \hfill \\ & \varphi (\omega ) = \arctan \left[ {\frac{U(\omega )}{{V(\omega )}}} \right] \hfill \\ \end{aligned} \right. $$

where: *A(ω)* is the amplitude, *1/A(ω)* is applied to express the dynamic stiffness of the rubber bushing; *φ(ω)* is the phase, which means the loss angle of the rubber bushing, the tangent of φ(ω) means the loss factor, and *ω* is the frequency.

11 $$ \frac{1}{A(\omega )} = \sqrt {\left( {\frac{{{\text{C}}_{{1}} {\text{K}}_{{2}} \left( {{\text{C}}_{{1}} {\text{ + C}}_{{2}} } \right)}}{{{\text{K}}_{1}^{3} {\text{C}}_{2} }}\omega } \right)^{2} + \left( {\frac{1}{{K_{1} }}} \right)^{2} } *\frac{1}{{\sqrt {1 - \left( {\left( {\frac{{(C_{1} + C_{2} )(C_{1} + C_{3} )}}{{C_{2} }} - \frac{{C_{1}^{2} }}{{K_{1} }}} \right)\frac{{{\text{K}}_{{2}} }}{{{\text{K}}_{1}^{2} }}\omega^{2} } \right)^{2} + \left( {\left( {\frac{{C_{1} + C_{3} }}{{K_{1} }} + \frac{{C_{1} + C_{2} }}{{C_{2} }}} \right)\omega } \right)}^{2} }} $$

12 $$ \varphi (\omega ) = - \arctan \left( {\frac{{(C_{1} + C_{3} ){\text{K}}_{{1}} + \left( {1 + \frac{{{\text{C}}_{{1}} }}{{{\text{C}}_{{2}} }}} \right)K_{1}^{2} }}{{K_{1}^{2} - \left[ {(C_{1} + C_{3} )\left( {1 + \frac{{{\text{C}}_{{1}} }}{{{\text{C}}_{{2}} }}} \right) - C_{1} } \right]\frac{{{\text{C}}_{{1}} }}{{{\text{K}}_{{1}} }}\omega }}} \right) + \arctan \left( {\frac{{(C_{1} + C_{2} )C_{1} K_{2} }}{{C_{2} K_{1}^{3} }}\omega } \right) $$

where: *1/A(ω)* represents the relationship between stiffness and frequency, *φ(ω)* represents the relationship between loss angle and frequency.

### Parameter identification

Parameter identification is mainly segmented into two parts: polynomial spring model identification and the FPM model identification. The main parameters of the polynomial spring model include *a*_*0*_, *a*_*1*_, *a*_*2*_, *a*_*3*_, and *a*_*4*_. The main parameters of the FPM model are K1, K2, C1,C2 and C3. In this paper, the stiffness’s and loss factor’s forecast data of rubber bushing under frequency 1–100 Hz are fitted and the parameters of the model are identified by least square method.

Least square method is a mathematical optimization technique to find the best function matching of data by minimizing the sum of squares of errors. The sum of squares of the errors between the unknown data easily obtained by using the least square method and the actual data can be minimized. More importantly, the least square method is usually applied for curve fitting.

The principle of the least square method is as follows:

A set of data (x_i_, y_i_) i = 1,2,……,n is applied. The fitting function is set to formula (13).

13 $$\mathop {\phi \left( {\text{x}} \right)}\limits ^{*} = a_{0} \phi_{0} (x) + a_{1} \phi_{1} (x) + \cdots + a_{n} \phi_{n} (x) = \sum\limits_{k = 0}^{n} {a_{k} \phi_{k} (x)} $$

According to the least square method:

14 $$ {\text{S}}(a_{{_{0} }} ,a_{{_{1} }} ,\ldots,a_{n} ) = \sum\limits_{i = 1}^{m} {\mathop {[a_{0} \phi_{0} (x_{i} ) + a_{1} \phi_{1} (x_{i} ) +\cdots + a_{0} \phi_{0} (x_{i} ) - y_{i} ]}\nolimits^{2} = \min } $$

Partial derivative of s in formula (14):

15 $$ \sum\limits_{i = 1}^{{\text{m}}} {\phi_{k} (x_{i} )[a_{0} \phi_{0} (x_{i} ) + a_{1} \phi_{1} (x_{i} ) + \cdots + a_{n} \phi_{n} (x_{i} ) - y_{i} ] = 0}  \quad (\kappa  = 0,1,2 \ldots \nu )$$

a_1_,a_2_,…,a_n_ are obtained by solving Eq. (15).

**Figure 18:**
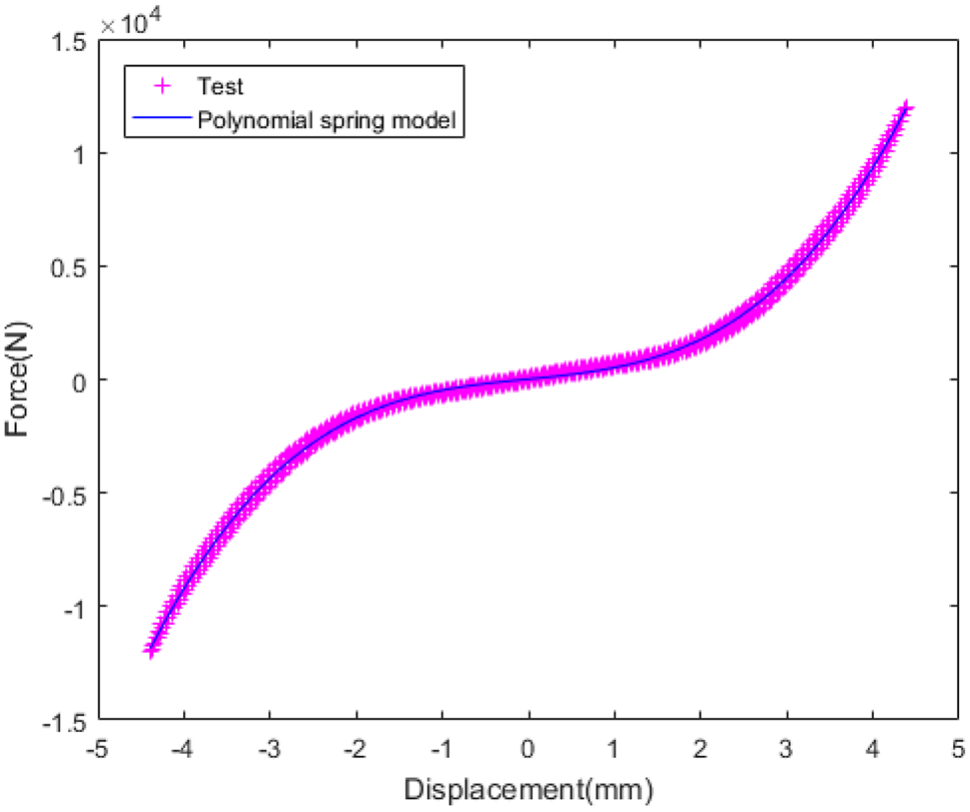
Fitting curve of static stiffness.

**Figure 19:**
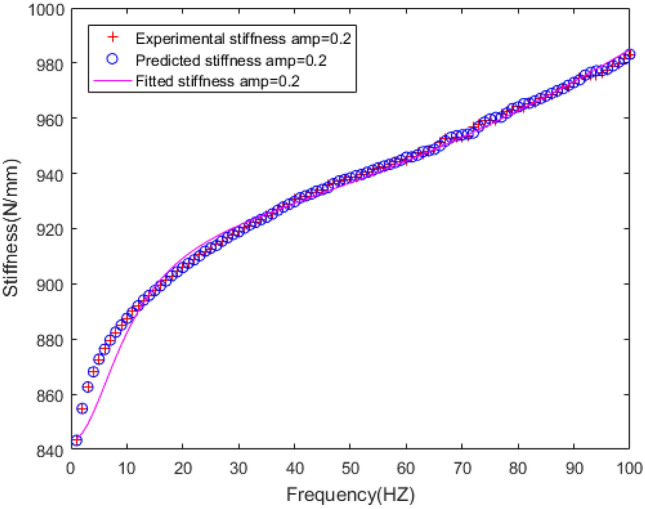
Fitting the curve of dynamic stiffness with five-parameter mathematical model under amplitude 0.2 mm.

**Figure 20:**
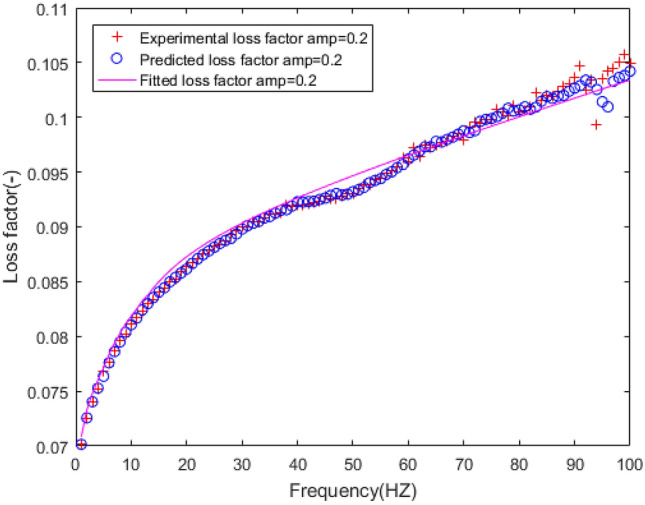
Fitting the curve of loss factor with five-parameter mathematical model under amplitude 0.2 mm.

**Figure 21:**
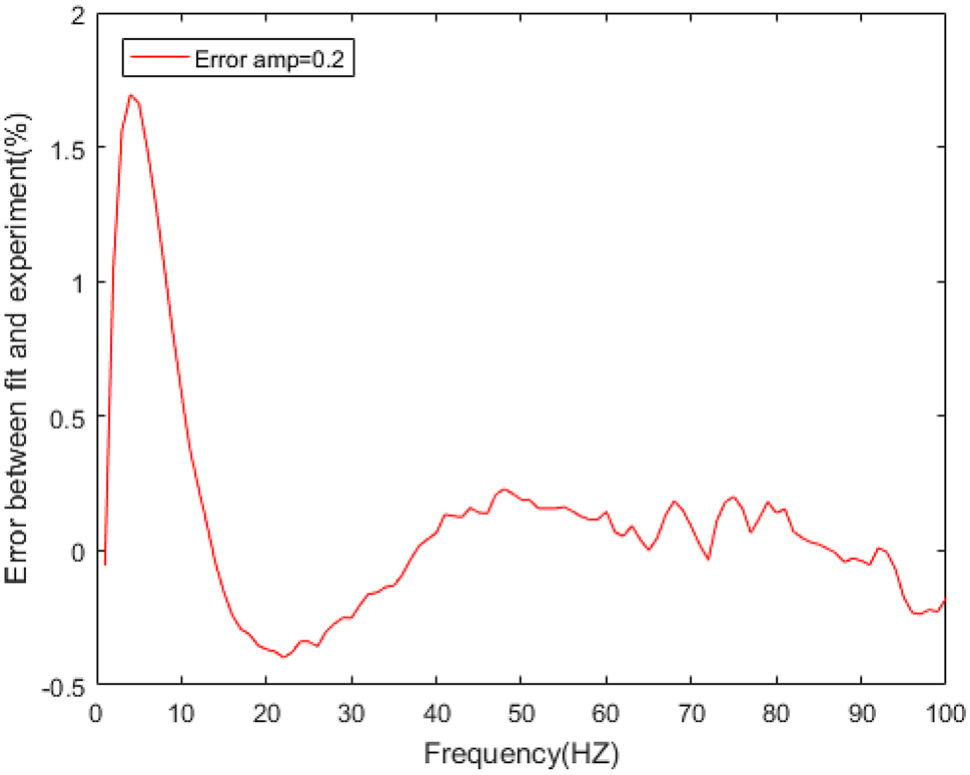
Errors of stiffness between five-parameter mathematical model and experiment under amplitude 0.2 mm.

**Figure 22:**
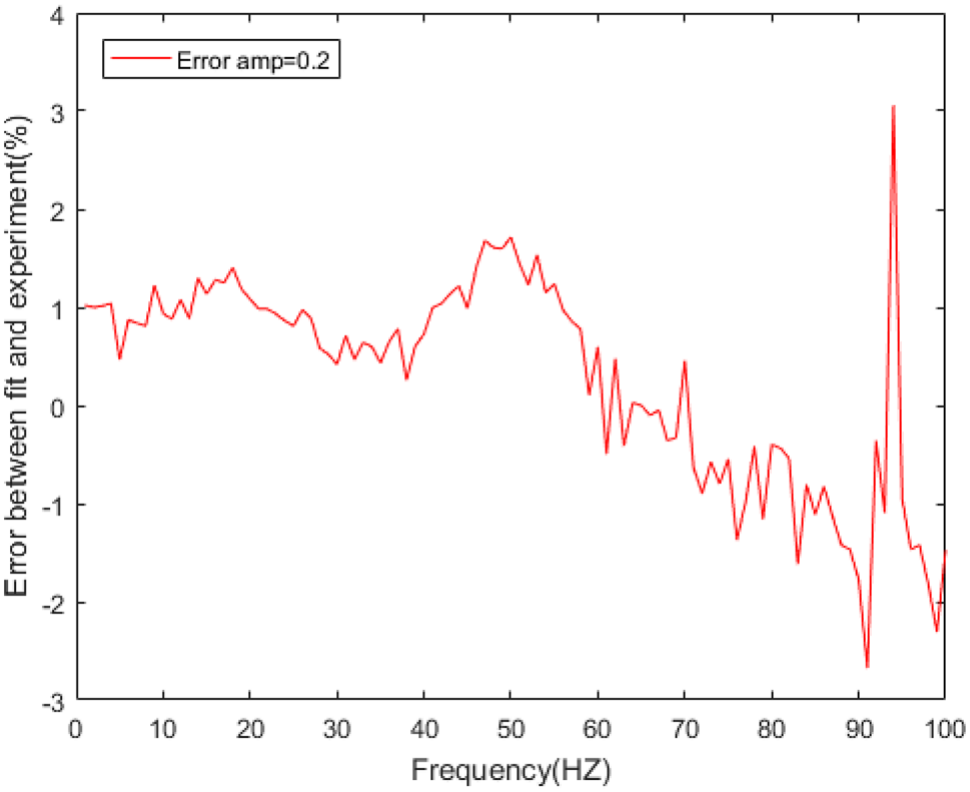
Errors of loss factor between five-parameter mathematical model and experiment under amplitude 0.2 mm.

The polynomial spring model and FPM model are identified by the least square method, and the fitting results are shown in the Figs. 18, 19, 20, 21, 22.

The correlation between force and load displacement is displayed in Fig. 18. The fitting results of the polynomial spring model and the test results are in good agreement. The force and slope increase with the rising loading displacement. In other words, with the rising loading displacement, the stiffness tends to increase. The identification parameters of the polynomial spring model are listed in Table 3.

**Table 3:** Identification parameters of polynomial spring model and FPM model.

Model	Parameters	Value
Polynomial spring model	a_0_	17.5057
	a_1_	380.6435
	a_2_	7.4051
	a_3_	121.0844
	a_4_	− 0.2597
Five-parameter mathematical model	K_1_	540.42
	K_2_	93.28845
	C_1_	4735.7545
	C_2_	− 6450.224
	C_3_	2.0269

From the perspective of vehicle ride comfort, the frequency range in Figs. 19 and 21 can be divided into three types: low frequency range (1–20 Hz), middle frequency range (21–80 Hz), high frequency range (81–100 Hz). It can be seen that the dynamic stiffness of the rubber bushing rises with the increase of frequency. In the low frequency range, the change of stiffness is non-linear, while in the middle and high frequency range, the variation of stiffness is approximately linear. The fitting results of the FPM model on forecast data are as follows: in the middle and high frequency range, the fitting error is less than 0.5%. The fitting error of dynamic stiffness is relatively large during 0 Hz to 10 Hz, but less than 2%. The fitting result of loss factor is presented in Figs. 20 and 22. The relationship between loss factor and frequency is similar to the relationship between dynamic stiffness and frequency. Due to insufficient rigidity of the test bench, when the frequency is over 90 Hz, the test data is fluctuated. Therefore, the fitting error of loss factor during 90 Hz to 100 Hz is larger compared with other frequencies, but less than 3%. In a conclusion, the FPM model can accurately describe the dynamic stiffness and loss factor of the rubber bushing. The identification parameters of the FPM model are listed in Table 3.

### Verifying the five-parameter mathematical model

**Figure 23:**
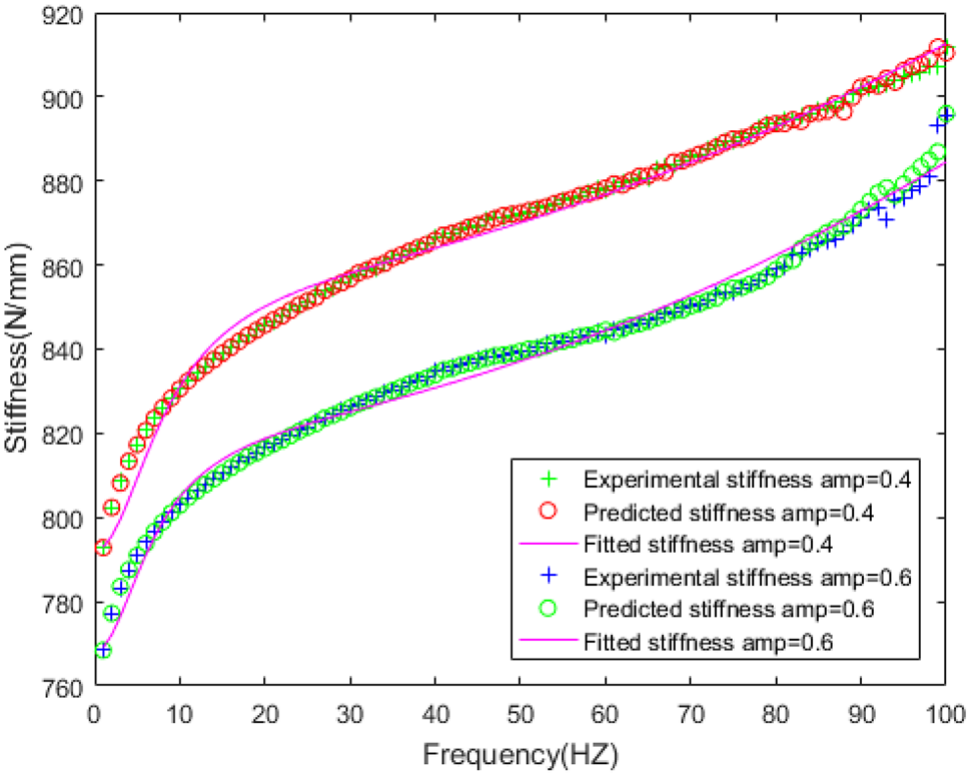
Fitting the curve of dynamic stiffness with five-parameter mathematical model under amplitude 0.4 mm, 0.6 mm.

**Figure 24:**
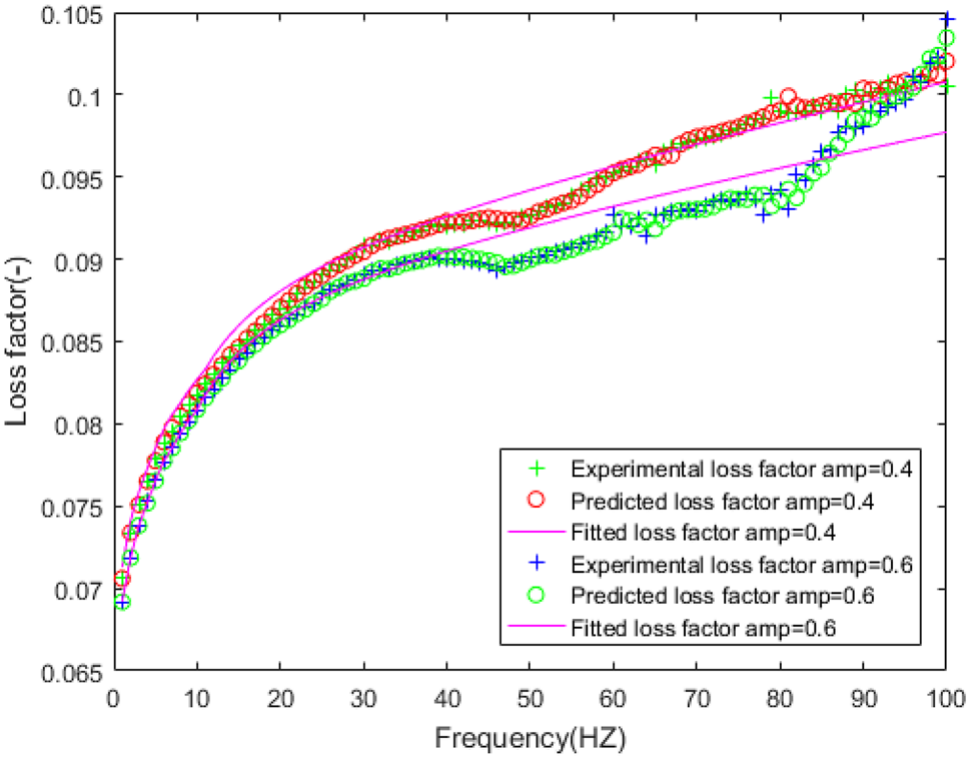
Fitting the curve of loss factor with five-parameter mathematical model under amplitude 0.4 mm, 0.6 mm.

**Figure 25:**
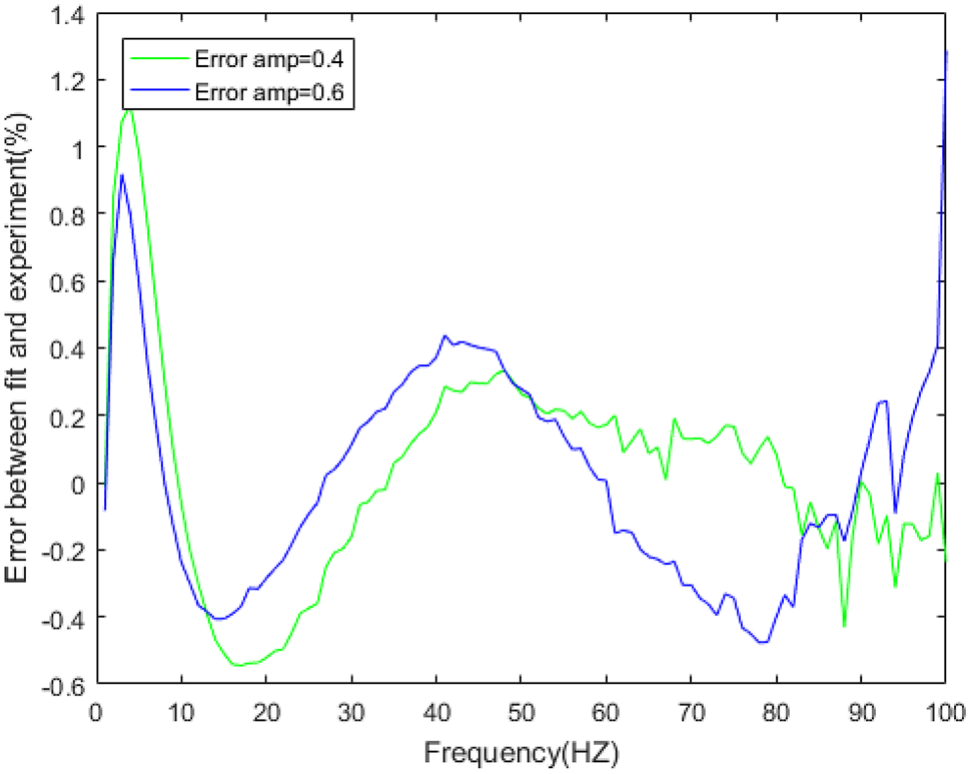
Errors of stiffness between five-parameter mathematical model and experiment under amplitude 0.4 mm, 0.6 mm.

**Figure 26:**
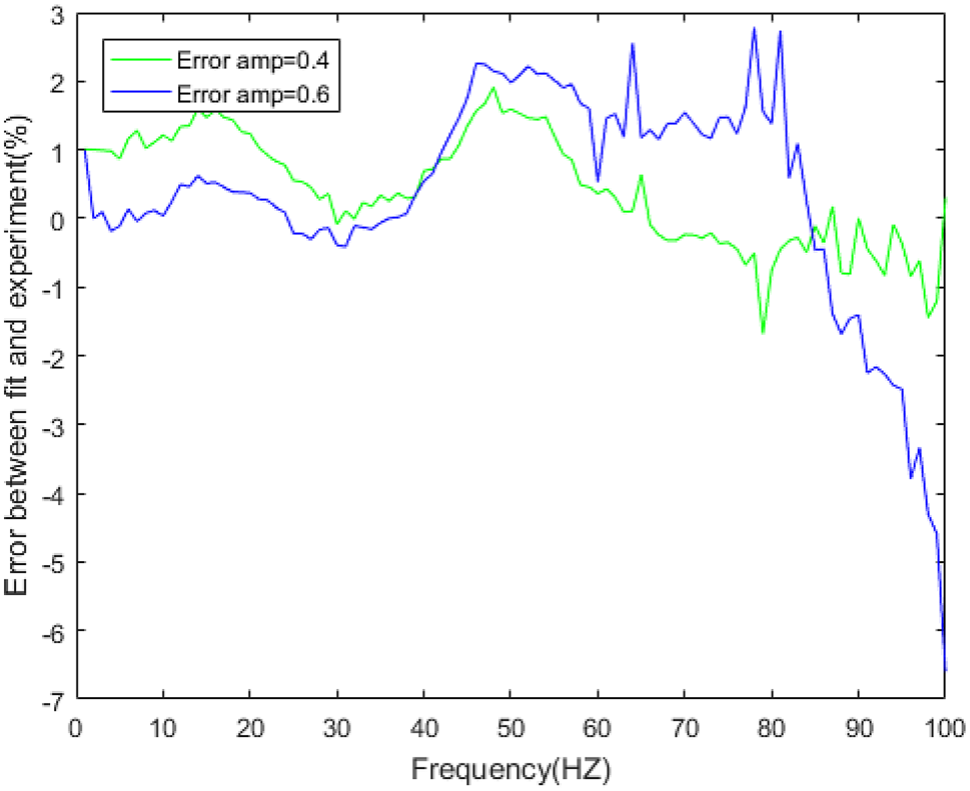
Errors of loss factor between five-parameter mathematical model and experiment under amplitude 0.4 mm, 0.6 mm.

In order to verify the feasibility of the FPM model, the expected dynamic stiffness and loss factor under amplitude 0.4 mm and 0.6 mm are fitted by FPM model, and the fitting error is analyzed in the Figs. 23, 24, 25, 26.

Figures 23, 24, 25, 26 illustrate the fitting results and error between forecast and fitting data under amplitude 0.4 mm and 0.6 mm. The fitting error of dynamic stiffness in the middle and high frequency range, the error is less than 0.5%. It is relatively large during 0 Hz to 10 Hz, but less than 1.2%. The fitting error of loss factor during 90 Hz to 100 Hz is larger than other frequencies, but less than 6%. Under amplitude 0.6 mm, the error of loss factor in high frequency range is mainly caused by insufficient stiffness of the test bench. The above results verify the feasibility of the five-parameter mathematical model.

## Conclusions

The GA-BP neural network is proposed to predict the frequency independence of rubber bushing. In order to apply the predicted results to the software for simulation, the FPM model is established to fit the forecast data on dynamic stiffness and loss factor, and the parameters of the five-parameter mathematical model are identified. According to the loading displacement and force, the relationship between dynamic stiffness and loss factor with frequency is derived under different amplitudes in this paper.

The results indicate that the dynamic stiffness and loss factor of the rubber bushing rise with the increase of frequency. In the low frequency range, the change is non-linear. In the middle and high frequency range, the variation is approximately linear. For the errors of dynamic stiffness and loss factor, the prediction errors of dynamic stiffness and loss factor are respectively less than 0.2% and 3%. And the fitting errors of dynamic stiffness and loss factor are respectively less than 2% and 3%.

According to the GA-BP calculation, the stiffness of the high frequency range can be predicted by the test data in the low frequency range. The algorithm not only reduces the test cycle time and cost, but avoids the test error due to insufficient rigidity of the test bench. The mechanical properties of rubber bushing are also affected by temperature, amplitude, and preload. The established GA-BP can be contributed to amplitude/preload/temperature correlation prediction. In addition, the proposed five-parameter mathematical model which accurately fitting of mechanical characteristics in high frequency range can be utilized to the software for simulation.

Future research should consider the temperature and preload correlation performance of rubber bushing, and apply the GA-BP neural network and five-parameter mathematical model to active suspensions to improve vehicle ride comfort and predict handling stability.
